# Sex determination through maxillary dental arch and skeletal base measurements using machine learning

**DOI:** 10.1186/s13005-024-00446-w

**Published:** 2024-08-30

**Authors:** Cristiano Miranda de Araujo, Pedro Felipe de Jesus Freitas, Aline Xavier Ferraz, Isabella Christina Costa Quadras, Bianca Simone Zeigelboim, Sidnei Priolo Filho, Svenja Beisel-Memmert, Angela Graciela Deliga Schroder, Elisa Souza Camargo, Erika Calvano Küchler

**Affiliations:** 1 School of Dentistry, Tuiuti University of Paraná, Curitiba, Paraná Brazil; 2 Graduate Program in Human Communication Health, Tuiuti University of Paraná, Curitiba, Paraná Brazil; 3 Center for Artificial Intelligence in Health – NIAS, Curitiba, Paraná Brazil; 4 Graduate Program in Dentistry, Orthodontics, Pontifícia Universidade Católica do Paraná, Curitiba, Brazil; 5 Graduate Program in Forensic Psychology, Tuiuti University of Paraná, Curitiba, Paraná Brazil; 6 Department of Orthodontics, University Hospital Bonn, Medical Faculty, Welschnonnenstr. 17, 53111 Bonn, Germany

**Keywords:** Maxilla, Sex characteristics, Artificial intelligence, Machine learning

## Abstract

**Background:**

Cranial, facial, nasal, and maxillary widths have been shown to be significantly affected by the individual’s sex. The present study aims to use measurements of dental arch and maxillary skeletal base to determine sex, employing supervised machine learning.

**Materials and methods:**

Maxillary and mandibular tomographic examinations from 100 patients were analyzed to investigate the inter-premolar width, inter-molar width, maxillary width, inter-pterygoid width, nasal cavity width, nostril width, and maxillary length, obtained through Cone Beam Computed Tomography scans. The following machine learning algorithms were used to build the predictive models: Logistic Regression, Gradient Boosting Classifier, K-Nearest Neighbors (KNN), Support Vector Machine (SVM), Multi-Layer Perceptron Classifier (MLP), Decision Tree, and Random Forest Classifier. A 10-fold cross-validation approach was adopted to validate each model. Metrics such as area under the curve (AUC), accuracy, recall, precision, and F1 Score were calculated for each model, and Receiver Operating Characteristic (ROC) curves were constructed.

**Results:**

Univariate analysis showed statistical significance (*p* < 0.10) for all skeletal and dental variables. Nostril width showed greater importance in two models, while Inter-molar width stood out among dental measurements. The models achieved accuracy values ranging from 0.75 to 0.85 on the test data. Logistic Regression, Random Forest, Decision Tree, and SVM models had the highest AUC values, with SVM showing the smallest disparity between cross-validation and test data for accuracy metrics.

**Conclusion:**

Transverse dental arch and maxillary skeletal base measurements exhibited strong predictive capability, achieving high accuracy with machine learning methods. Among the evaluated models, the SVM algorithm exhibited the best performance. This indicates potential usefulness in forensic sex determination.

**Supplementary Information:**

The online version contains supplementary material available at 10.1186/s13005-024-00446-w.

## Introduction

The identification of human remains, even in extreme situations such as natural disasters or man-made causes, is crucial for humanitarian and legal reasons, enabling the establishment of a demographic profile in the identification process [1, 2]. Determining sex from osteological and dental evidence has been a relevant interdisciplinary field of study in various specialties, both in forensic and archaeological contexts [2]. The study of anatomical characteristics of bones, such as ancestry, sex, and age, can aid in the process of determining individuals’ identities, acting as a secondary part of identification, with craniofacial morphology providing satisfactory results in sex determination [1, 3].

The bones of the head and neck, as well as teeth, are important in the forensic context of identification, mainly due to their resistance to high temperatures [4]. Methods for determining sex through cranial analysis in adults are already well-established and demonstrate high accuracy [5]. Cranial, facial, nasal, and maxillary widths have been shown to be significantly affected by the individual’s sex [6]. Additionally, dental arch width emerges as a relevant indicator for individual identification and sex determination [7]. For morphometric analysis of these regions, computed tomography and magnetic resonance imaging provide valuable information about anatomy, preserving the relationship between structures in space, and are becoming increasingly common in forensic investigations [4].

In the forensic field, machine learning algorithms have played a significant role. This subfield of artificial intelligence allows performing predictions without the need for explicit programming. Mathematical models are developed from sets of training data samples [8]. This technique focuses on creating algorithms capable of learning from data and making predictions based on them. One of the most frequently performed tasks in machine learning is classification, in which the algorithm learns to label individuals according to the classes to be predicted, based on specific characteristics learned from the data [9]. Computed tomography has shown great importance in the forensic field. In this context, the use of morphometric measurements of the mandible, maxillary sinus, temporal bone, and dental measurements, assessed by Cone-Beam Computed Tomography (CBCT) and analyzed with various machine learning algorithms, has been widely reported in the scientific literature, demonstrating promising results [10–13].

To the best of our knowledge, no studies measured dental arch and maxillary base for sex determination using machine learning techniques. Therefore, the present study aims to use measurements of dental arch and maxillary skeletal base to determine sex, employing supervised machine learning. The null hypothesis for this study is that there is no significant difference in the predictive accuracy of sex determination when using dental arch and maxillary skeletal base measurements compared to random predictions.

## Methods

### Study design and setting

This cross-sectional study investigated 100 patients undergoing maxillary and mandibular tomographic examinations at a dental computed tomography service located in southern region of Brazil. The research was conducted in accordance with the principles established in the Helsinki Declaration and received approval from the Research Ethics Committee of Tuiuti University of Paraná, Brazil (Approval Number: 6.305.456), on September 17, 2023. Informed consent was obtained from all adult patients and from the parents or legal guardians of minor patients participating in the study.

### Participants

The following inclusion criteria were established: participants should not have previous orthodontic treatment; no missing upper teeth, except for third molars; and the first premolars should be erupted. Patients with facial malformations, a history of severe facial trauma, or bone lesions affecting the dimensions of the bones were excluded from the sample.

To ensure a better comparison between genders, measurements were performed on a matched sample in a 1:1 ratio regarding gender and age.

### Variables and measurements

The CBCT were performed using the 3D Axeos equipment from Dentsply Sirona (Dentsply Sirona Inc, New York, USA). The acquisition volume covered an area of 17 × 13 cm, with a voxel size of 0.16 mm. A specialized examiner with a master’s degree in Dental Radiology conducted image interpretation and measurements. The images were presented randomly for evaluation.

**Fig. 1:**
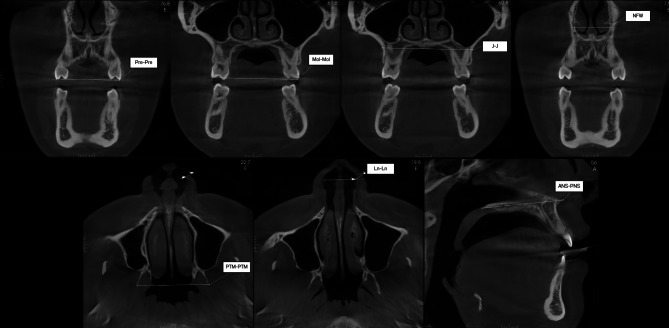
Landmarks for measurements of the dental arch and maxillary skeletal base

The predictor variables used, collected from the CBCT exams, include the inter-premolar width (Pre-Pre), which is the distance between the tips of the vestibular cusps of the upper first premolars; the inter-molar width (Mol-Mol), which measures the distance between the mesio-vestibular cusps of the upper first molars; and the maxillary width (J-J), which is the distance between the jugal points. Additionally, the inter-pterygoid width (PTM-PTM) was considered, which is the distance between the lateral plates of the pterygoid processes of the sphenoid bone. The nasal cavity width (NFW) was measured between the most lateral points of the nasal cavity, and the nostril width (Ln-Ln) was determined by the distance between the most lateral points of the nostrils. Finally, the maxillary length (ANS-PNS) was assessed by the distance between the anterior and posterior nasal spines. The examiner selected the image slice that exhibited the greatest dimension of the structure to be evaluated. The landmarkers and measurements are showed in the Fig. 1.

Aiming to ensure adequate intra-examiner measurement reproducibility, ten measurements were randomly selected and re-evaluated after one week, yielding a Dahlberg error value of less than 1%, demonstrating the reliability of the assessments.

### Data analysis and model construction

To select the predictor variables with the highest impact for inclusion in the supervised machine learning model, a univariate analysis was conducted using the statistical software JASP version 0.19.0, employing Student’s t-test for independent samples. This preliminary analysis aimed to identify significant differences between sexes before building the models. The power analysis for each comparison was calculated using the statistical software GPower version 3.1.9.6, while effect size was determined by calculating Cohen’s d. A significance level of 10% (α = 0.10) was adopted to allow the inclusion of any relevant variable for the model. To determine the cutoff values and identify the threshold at which each variable is classified as male or female, Receiver Operating Characteristic (ROC) curves were generated for each variable.

For the construction of predictive models, the following supervised machine learning algorithms were used: Logistic Regression, Gradient Boosting Classifier, K-Nearest Neighbors (KNN), Support Vector Machine (SVM), Multi-Layer Perceptron Classifier (MLP), Decision Tree, and Random Forest Classifier. To optimize the model’s performance and find the best combination of hyperparameters, the Grid Search method was employed. This method involves the systematic evaluation of predefined combinations of hyperparameters. All algorithms were developed using the Python programming language (version 3.10.12) in the Google Colab environment, utilizing the ‘scikit-learn’ library. The programming code used is available as open access (DOI: 10.5281/zenodo.10988064).

### Training, cross-validation, and test

**Fig. 2:**
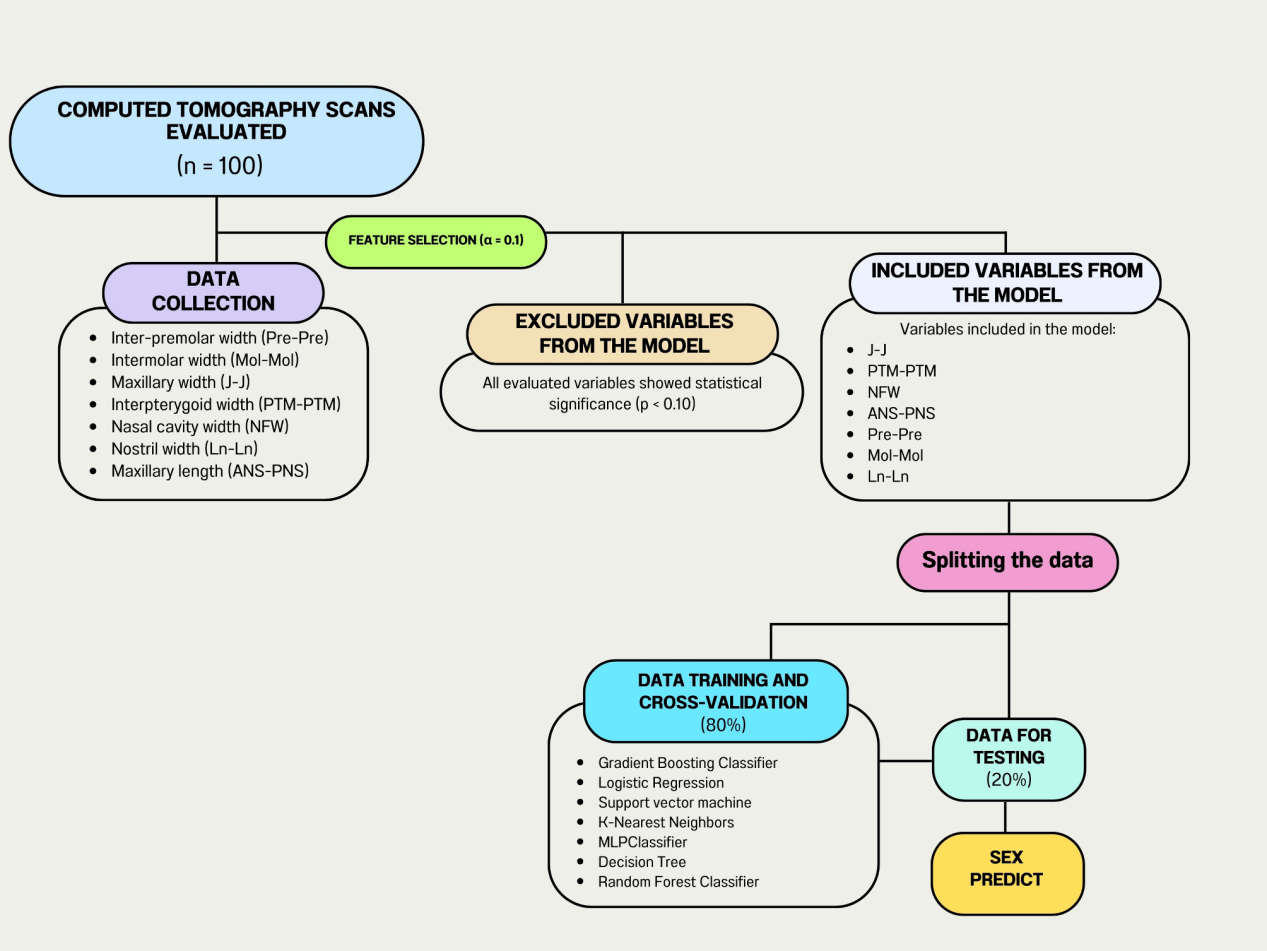
Flowchart diagram illustrating the data analysis process using machine learning models

The data was divided into training and cross-validation sets (80%) and test data (20%). The test data was exclusively reserved to evaluate the predictive capacity of the model. This division was performed using the ‘train_test_split’ function from the ‘sklearn.model_selection’ library. Additionally, the data was normalized using the standardization technique before training and testing the models. This ensures that all features have a comparable scale, essential for algorithms sensitive to data scale. The cross-validation technique was then employed to estimate how well the model is able to generalize to unseen data, by dividing the data into k subsets and training the model k times. In each iteration, k-1 subsets were used for training, while the remaining data was reserved for validation. Thus, an average estimate of the validation performance was calculated based on a 10-fold cross-validation (Fig. 2).

### Metrics and model evaluation

To evaluate the models’ ability to distinguish between different classes, ROC curves were generated and the areas under the curve (AUC) were calculated for each. To do this, false positive and true positive rates were determined at various classification thresholds using the ‘roc_curve’ function from the ‘scikit-learn’ package, with the actual labels from the test set and the predicted probabilities for the positive class from the model. The AUC metric was then obtained through the ‘roc_auc_score’ function, also from the ‘scikit-learn’ package, providing a quantitative measure of the model’s discriminatory power. ROC curves were plotted using the ‘matplotlib.pyplot’ library, allowing for a detailed assessment of each model’s performance in the binary classification task.

Additionally, metrics such as accuracy, recall, precision, and F1 Score were calculated for each model. The feature importance evaluation function from the Scikit-learn library was employed to visually identify the most relevant variables in each model formulation, aiming to understand which features have the greatest influence on the model’s predictive capacity. However, this evaluation was not conducted for the KNN, SVM, and MLP models due to the peculiarities of these algorithms, which do not support this function.

## Results

A total of 100 patients were included in the sample, equally divided between men and women, with an age range from 10 to 88 years and a mean age of 38.5 ± 20.6 years (38.2 ± 19 for men and 38.8 ± 22.3 for women). There was no significant difference in age between the groups (*p* = 0.883). Univariate analyses revealed that all skeletal and dental variables showed statistical significance (*p* < 0.10). Only one variable had a power below 0.7, indicating that most variables had adequate power for the evaluated sample size. The NFW variable had the smallest effect size, with a p-value close to the stipulated significance level. Consequently, all variables were used for training the supervised machine learning model (Table 1). The ROC curves and cutoff points, identifying the thresholds at which each variable is classified as male or female, can be viewed in Additional File 1.

**Fig. 3:**
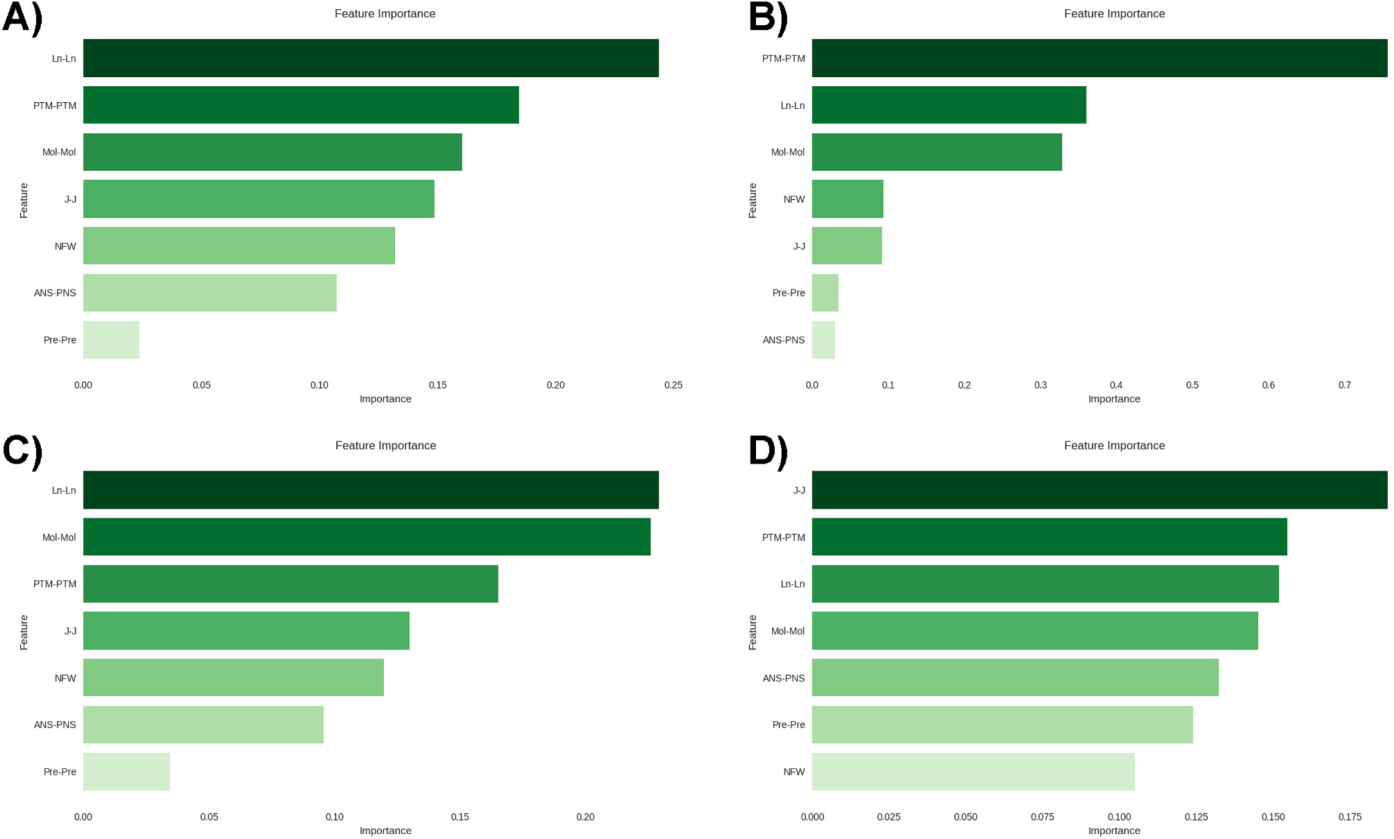
Results of feature importance analysis from four machine learning models. **A** – Gradient Boosting Classifier, **B** – Logistic Regression, **C** – Decision Tree, **D** – Random Forest Classifier

**Fig. 4:**
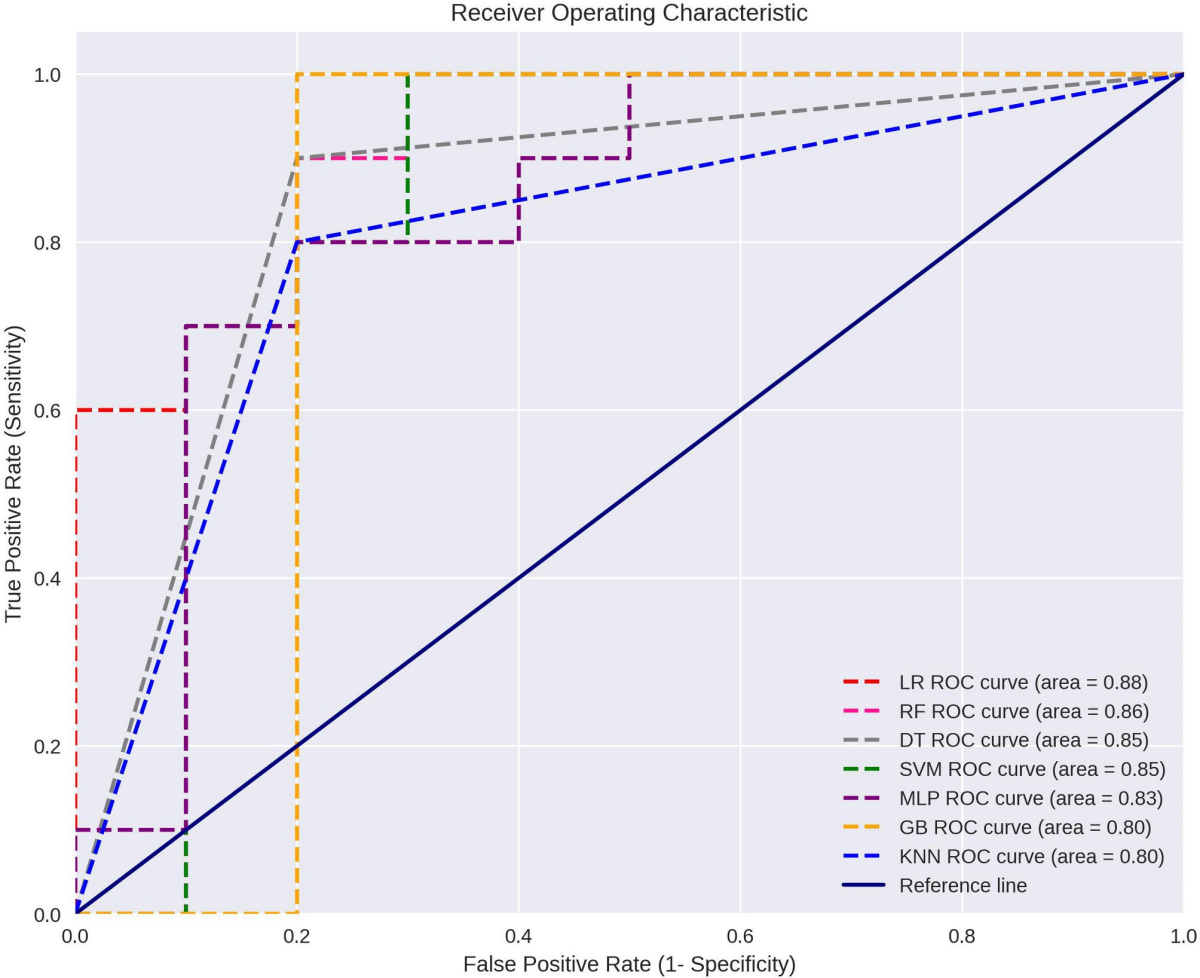
Evaluation of Classification Models using ROC Curves. LR – Logistic Regression, SVM – Support Vector Machine, KNN – K-Nearest Neighbors, GB – Gradient Boosting, MLP – Multilayer Perceptron, RF – Random Forest, DT – Decision Tree

**Fig. 5:**
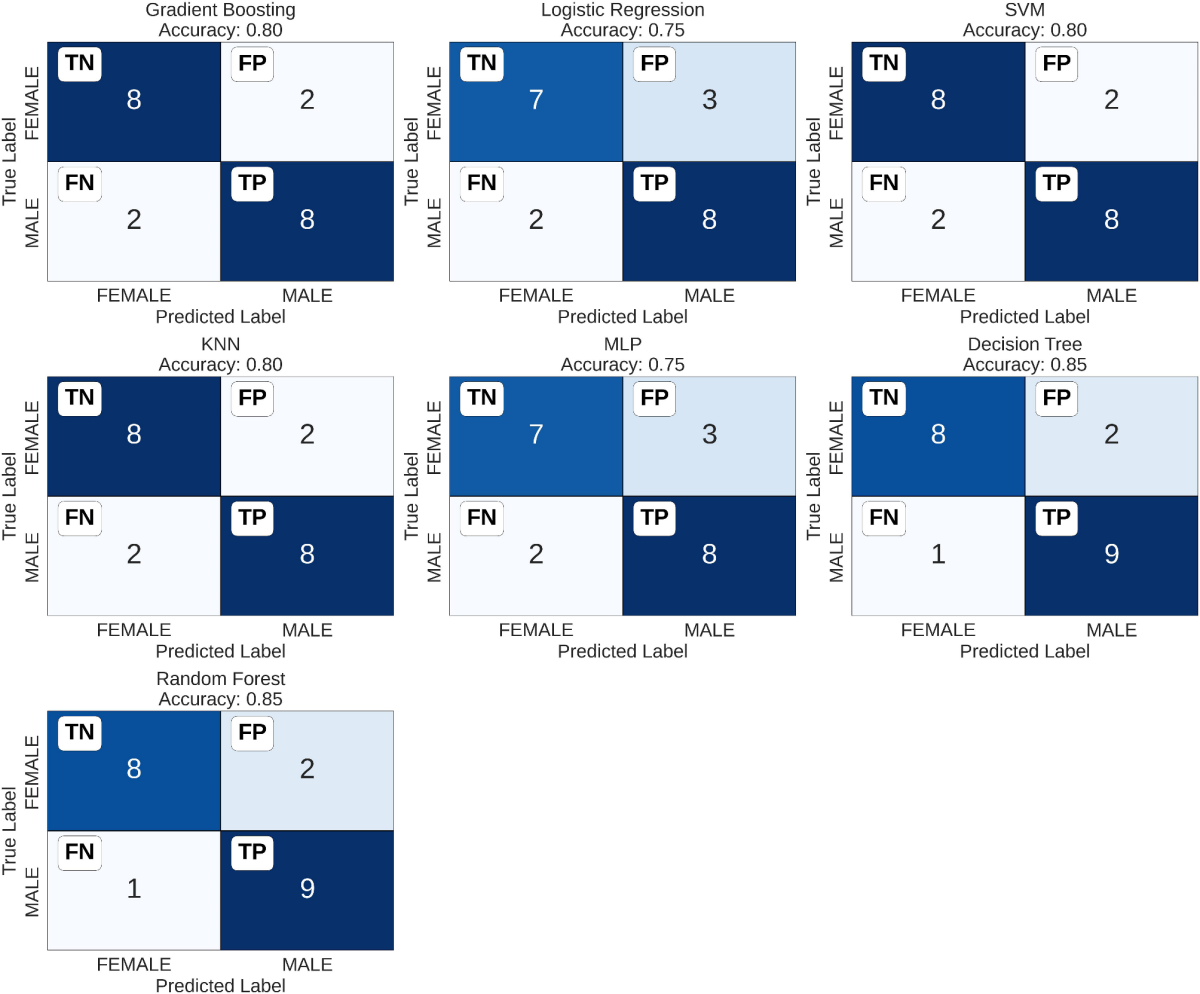
Confusion matrix for the test data, for all tested machine learning models

Thus, a total of five skeletal and two dental variables were included to train the machine learning predictive model. The Ln-Ln variable demonstrated greater importance in two of the constructed models, while the Mol-Mol variable stood out among the dental measurements (Fig. 3). The performance of all trained predictive models, along with the respective best hyperparameters for each model are shown in Table 2. When considering the models’ ability to distinguish between different classes, assessed by AUC, the models ranged from 0.80 to 0.88 (Fig. 4). The highest values were observed for logistic regression, random forests, decision tree, and SVM models, with the latter showing the least disparity in metrics between cross-validation and test data. The confusion matrix for each tested algorithm, based on the test data, is presented in Fig. 5.

**Table 1:** Maxillary measurements by sex

Measurement	*n*	Mean ± SD	*p*-value*	Effect size**	Power test
PTM-PTM					
Male	50	51.6 ± 6.91	0.003	0.616	0.86
Female	50	47.9 ± 4.80			
J-J					
Male	50	60.9 ± 5.24	< 0.001	0.727	0.94
Female	50	57.3 ± 4.79			
Ln-LN					
Male	50	30.1 ± 6.45	0.009	0.535	0.77
Female	50	27.0 ± 4.82			
NFW					
Male	50	22.7 ± 4.96	0.098	0.326	0.33
Female	50	21.1 ± 4.75			
ANS-PNS					
Male	50	54.2 ± 4.35	0.012	0.510	0.70
Female	50	52.2 ± 3.52			
Pre-pre					
Male	50	47.0 ± 6.25	0.010	0.527	0.75
Female	50	44.4 ± 2.97			
Mol-Mol					
Male	50	56.7 ± 5.49	< 0.001	0.741	0.96
Female	50	53.4 ± 3.09			

Legends * p-value of the Student’s T test for independent samples

** Effect size calculated by Cohen’s d

**Table 2:** Summary of metrics obtained for the cross-validation and test stages of the models, along with their respective optimal hyperparameters

Model	Optimal hyperparameters (random_state = 24)	Cross-validation results (cv = 10)	Test data results
Logistic Regression	C: 0.3	Accuracy = 0.63	Accuracy = 0.75
	max_iter: 50	Precision = 0.62	Precision = 0.75
	penalty: l1	Recall = 0.62	Recall = 0.75
	l1_ratio: 0.2	F1-Score = 0.62	F1-Score = 0.75
	solver: liblinear		
Gradient Boosting Classifier	n_estimators: 2000	Accuracy = 0.59	Accuracy = 0.80
	learning_rate: 0.01	Precision = 0.59	Precision = 0.80
	criterion: friedman_mse	Recall = 0.59	Recall = 0.80
	max_depth: 5	F1-Score = 0.59	F1-Score = 0.80
	loss: deviance		
K-Nearest Neighbors	n_neighbors: 1	Accuracy = 0.75	Accuracy = 0.80
	weights: uniform	Precision = 0.75	Precision = 0.80
	leaf_size: 1	Recall = 0.75	Recall = 0.80
	p: 5	F1-Score = 0.75	F1-Score = 0.80
Support Vector Machine	kernel: rbf	Accuracy = 0.78	Accuracy = 0.80
	C: 0.9	Precision = 0.78	Precision = 0.80
	gamma: auto	Recall = 0.78	Recall = 0.80
		F1-Score = 0.77	F1-Score = 0.80
MLP Classifier	activation: relu	Accuracy = 0.73	Accuracy = 0.75
	alpha: 1.0	Precision = 0.72	Precision = 0.75
	hidden_layer_sizes: 1000	Recall = 0.72	Recall = 0.75
	learning_rate_init: 0.1	F1-Score = 0.73	F1-Score = 0.75
	max_iter: 50		
	solver: sgd		
Decision Tree	criterion: entropy	Accuracy = 0.66	Accuracy = 0.85
	max_depth: none	Precision = 0.66	Precision = 0.85
	splitter: best	Recall = 0.66	Recall = 0.85
		F1-Score = 0.66	F1-Score = 0.85
Random Forest Classifier	max_depth: 10	Accuracy = 0.70	Accuracy = 0.85
	n_estimators: 200	Precision = 0.70	Precision = 0.85
	min_samples_split: 2	Recall = 0.70	Recall = 0.85
	min_samples_leaf: 1	F1-Score = 0.70	F1-Score = 0.85
	criterion: gini		
	max_features: auto		

## Discussion

Sex determination and identification of population origin are two essential elements in forensic investigation, being fundamental tasks when dealing with human skeletal remains [14, 15]. Therefore, the aim of this study was to employ measurements of the dental arch and maxillary skeletal base to determine sex, using supervised machine learning. The construction of this model aims to enhance sex determination based on the maxillary complex, using features extracted from CBCT images. Although other craniofacial measurements have been used for sexual dimorphism assessment, such as the frontal bone, dental measurements, mandibular measurements, hyoid bone, and cervical vertebrae [4, 16–20], until now, no studies have been found in the literature employing maxillary measurements for sex determination using machine learning algorithms.

Machine learning involves the use of various mathematical models capable of generating predictive models through data analysis [20]. Developing a model to estimate sex is based on solving a classification task, which is a common task in machine learning [9]. One of the algorithms used for this task is Support Vector Machine (SVM), a kernel-based machine learning model employed for classification and regression tasks. The primary goal of the SVM algorithm is to find a hyperplane that maximizes the margin between different classes in the dataset [21]. SVM has proven effective in sex estimation based on cranial measurements, being considered one of the best-performing predictive models in forensic literature [8, 9, 22]. In this study, the SVM-based predictive model demonstrated the best performance during both the testing and cross-validation stages.

The presence of sexual dimorphism in the maxillary complex has been observed in several studies [6, 23, 24], demonstrating good predictive capacity in sex determination. Similarly, interdental transverse dimensions in the maxillary arch show dimorphic traits, in which it is larger in males than females [25, 26]. Among the evaluated measurements, skeletal dimensions showed good predictive power, while interdental dimensions also demonstrated a high effect size, with the intermolar distance exhibiting the largest effect among all evaluated dental variables. The NFW variable was the only one that showed a test power below 0.7. Although it is possible to hypothesize that a larger sample size could increase the observed test power, the vast majority of variables showed adequate power, thus validating the sample size used. Additionally, despite intermolar distance (Mol-Mol) showing the largest effect size, the Ln-Ln variable was the one that showed greater importance for most of the trained models, in which it was possible to estimate the importance of the variables. Extrapolating to the forensic context, skeletal characteristics can be considered more stable, since teeth may be subject to orthodontic movement, as well as prosthetic rehabilitations.

In general, females have lower bone mass compared to males, regardless of the age range assessed [27]. This sexual dimorphism becomes more evident due to changes in bone tissue caused by sex hormones (estrogens and androgens), genetics, and inflammatory processes, which affect the formation, resorption, and death of osteoclasts, making it a multifactorial process [4, 27]. Most of the bone and muscle mass is acquired before the age of 18. Sex hormones and the GH/IGF-1 axis regulate bone growth in both sexes, resulting in skeletal differences during puberty. As a result, men tend to have longer and wider bones due to differences in periosteal apposition and endosteal resorption, although bone mineralization is similar between the sexes [28]. This difference is also reflected in skull size [29]. In this regard, the univariate analysis of the assessed variables revealed statistical significance, demonstrating evident differences in maxillary measurements between men and women.

The representativeness of a sample in relation to a target population occurs when the estimates obtained, or their interpretation can be generalized to that target population. In the context of sexual dimorphism, it is important to consider factors such as the age range of individuals in the sample and the individual characteristics of each region. When examining the age of participants, statistically significant differences between sexes in the transverse widths of the maxillary skeleton were identified in age groups between 10 and 14 years, with male individuals generally showing greater incremental growth changes than female individuals. Additionally, the morphological characteristics of dental arches may vary according to ethnicity [6, 25]. In the present study, the age range of participants was from 10 to 88 years and was matched between groups in a 1:1 ratio to prevent age from acting as a confounding factor in the analysis. Although the inclusion of a broader age range may reduce the accuracy of the model, it also increases the external validity of the study, thus providing better applicability in the forensic context. In forensic settings, accurately determining age can be challenging due to the variability in the quality of available structures. While this decision might have decreased the precision of the models, it enhanced the external validity and applicability of our findings, making them more generalizable. Regarding ethnicity, it is important to mention that the data collection was carried out in the South region of Brazil, which is characterized by its unique miscegenation. Therefore, the sample consisted exclusively of Brazilians, which limits its representativeness. Therefore, it is recommended to conduct studies in other ethnic groups for a more comprehensive understanding of sexual dimorphism in these structures.

The bones of the head and neck are primarily originate from the branchial arches and develop around the fourth week of gestation [30]. As a result of this process, it is common for the structures of the male maxilla to be larger than those of the female maxilla in almost all dimensions, especially in length and total width of the maxilla [6, 23]. In the present study, measurements of the dental arch and maxillary skeletal base showed good predictive capacity for identifying sex. Additionally, the use of metric parameters was an approach adopted to minimize subjectivities, with all measurements performed in CBCT scans, which proved to be an excellent way to identify precise measurements, even considering the overlap of some bone structures. Although the variables used showed good predictive power, it is reasonable to suppose that even greater accuracy could be achieved with a larger sample size, and individuals from different ethnicities should be included in future studies. However, the results of the present study highlight the predictive potential of these variables, which are important markers for sexual dimorphism and may have relevant applicability in forensic science.

## Conclusion

The transverse measurements of the maxillary dental arch and measurements of the maxillary skeletal base demonstrated good predictive capacity, showing robust accuracy values using machine learning techniques. Among the evaluated models, the SVM algorithm exhibited the best performance. This suggests promising applicability in sex determination in forensic contexts. The analysis of the data rejected the null hypothesis, as the results indicate a difference in predictive accuracy for sex determination using dental arch and maxillary skeletal base measurements compared to random predictions.

### Electronic supplementary material

Below is the link to the electronic supplementary material.

Supplementary Material 1: Additional file 1 ROC curves and cutoff values for each variable, identifying the thresholds at which the variables are classified as male or female

## Data Availability

No datasets were generated or analysed during the current study.
