# Supplementary figures and images for: Sex determination through maxillary dental arch and skeletal base measurements using machine learning
Source: Head Face Med. 2024 Aug 30;20:44. doi: 10.1186/s13005-024-00446-w (PMC11363530; doi:10.1186/s13005-024-00446-w)

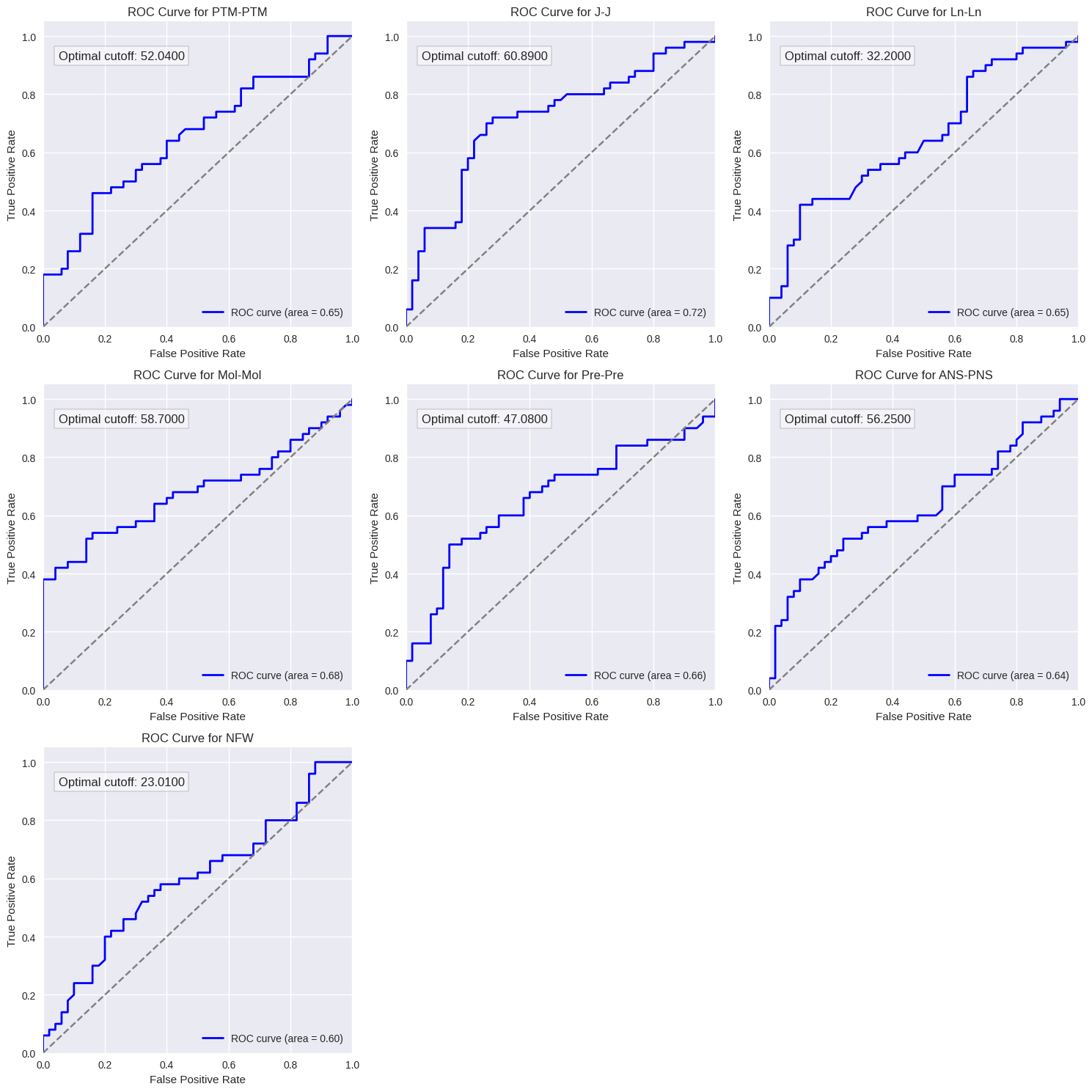

Supplement: Supplementary file 1 — Supplementary Material 1: Additional file 1 ROC curves and cutoff values for each variable, identifying the thresholds at which the variables are classified as male or female [file 13005_2024_446_MOESM1_ESM.jpg]
